# α-Tocopherol Transfer Protein-Null Mice with Very Low α-Tocopherol Status Do Not Have an Enhanced Lipopolysaccharide-Induced Acute Inflammatory Response

**DOI:** 10.1016/j.cdnut.2022.100017

**Published:** 2023-01-13

**Authors:** Megumi Hashida, Katherine M. Ranard, Andrew J. Steelman, John W. Erdman

**Affiliations:** 1 Division of Nutritional Sciences, University of Illinois at Urbana-Champaign, Urbana, IL, USA; 2 Department of Pediatrics, Section of Developmental Biology, University of Colorado Anschutz Medical Campus; Aurora, CO, USA; 3 Department of Animal Sciences, University of Illinois at Urbana-Champaign, Urbana, IL, USA; 4 Department of Food Science and Human Nutrition, University of Illinois at Urbana-Champaign, Urbana, IL, USA

**Keywords:** antioxidant, vitamin E, *RRR*-α-tocopherol, adolescent, *Ttpa*-null mouse, lipopolysaccharide

## Abstract

**Background:**

The α-tocopherol transfer protein-null (*Ttpa*^*−/−*^) mouse model is a valuable tool for studying the molecular and functional consequences of vitamin E (α-tocopherol, αT) deficiency. Because αT has been associated with reduced oxidative stress and improved immune function, we hypothesized that depleted αT concentration would exacerbate LPS-induced acute inflammatory response in the brain and heart of *Ttpa*^*−/−*^ mice fed a vitamin E deficient (VED) diet.

**Objectives:**

The objective was to investigate how extremely low αT status, followed by exposure to LPS, altered the acute inflammatory response to LPS in *Ttpa*^*−/−*^ and wild-type (*Ttpa*^*+/+*^) mice.

**Methods:**

Three-week-old male *Ttpa*^*+/+*^ and *Ttpa*^*−/−*^ littermates (*n* = 36/genotype) ingested a VED diet ad libitum for 4 wk. At week 7, mice received an intraperitoneal LPS (1 or 10 μg/mouse) or saline (control) injection and were killed 4 h postinjection. Brain and heart IL-6 protein concentrations and tissue and serum αT concentrations were measured via ELISA and HPLC with photodiode array detection, respectively. Hippocampal *Il-6*, *Tnf*, and *Gpx1* gene expression were measured via reverse transcriptase-quantitative polymerase chain reaction, and blood immune cell profiles were measured via a hematology analyzer.

**Results:**

αT accumulation in analyzed tissues and serum of *Ttpa*^*−/−*^ mice was substantially lower than *Ttpa*^*+/+*^ mice. Circulating white blood cell concentration, particularly lymphocytes, were lower in all LPS groups compared with controls (*P* < 0.01). The 10 μg LPS groups had elevated IL-6 in the cerebellum and heart compared with controls, confirming an acute inflammatory response (*P* < 0.01). Hippocampal and heart *Il-6* gene expression in the LPS-treated *Ttpa*^*−/−*^ mice was upregulated in a dose-dependent manner (*P* < 0.05).

**Conclusions:**

The 10 μg LPS dose enhanced inflammatory markers in the brain, heart, and serum in each genotype but the lower αT status in *Ttpa*^*−/−*^ mice did not further impact the acute immune responses.

## Introduction

Oxidative stress is associated with many abnormalities, such as lipid peroxidation, DNA and protein damage, inflammation, and neurodegeneration. Vitamin E (α-tocopherol, αT) can prevent lipid peroxidation and oxidative stress in cell membranes owing to its antioxidant function [[1], [2]]. αT insufficiency may enhance oxidative stress in the brain, increase susceptibility to neurological decline, and perturb the immune system [[3], [4], [5]]. Several studies have shown that severe vitamin E deficiency in humans and experimental animals causes neurological pathologies, such as ataxia and peripheral neuropathy [[3], [4], 6]. αT may be particularly essential for brain specific cell types, such as Purkinje neurons in the cerebellum, and for lipid-rich myelin, which wraps around axons and increases signal transmission speed [3]. The cerebellar αT turnover rate is more rapid than the other brain regions [7], and α-tocopherol transfer protein (α-TTP), an important vitamin E regulatory protein, is highly expressed in the cerebellum compared with the other brain regions [8]. Local α-TTP, specifically in the cerebellum, may help the regulation of αT homeostasis in the central nervous system (CNS).

Immune cells contain higher vitamin E concentrations than red blood cells, and vitamin E may be a critical component for the regulation of immune function [9]. Vitamin E plays a role in lymphocyte proliferation, helper T cell activity, and IL-2 production in mice [[10], [11], [12]] and enhances immune function, including natural killer cell activity and phagocytic ability of macrophages in lungs of rats [13]. Neutrophils from preterm infants with vitamin E deficiency showed exacerbated bactericidal activity and phagocytic capacity [14]. Furthermore, vitamin E has been reported to regulate gene expression profiles of T cells with/without anti-CD3/anti-CD28 stimulation related to cell cycle, Th1/Th2 balance, and cytokine production in mice [15]. The effects of vitamin E deficiency on abnormal immune function, such as stimulated cytokine production and impaired T cell proliferation, can be reversed by vitamin E supplementation [16]. Finno et al. [5, 17] indicated that α-TTP knockout *(Ttpa*^*−/−*^) mice induce enhanced oxidative stress response and exacerbate gene expression related to the innate immune response. Therefore, vitamin E status appears to impact both the innate and adaptive immune systems.

Identifying the effects of vitamin E depletion in the body is essential for understanding its tissue-specific roles. However, modeling vitamin E deficiency in animals is challenging. Depleting αT in the brains of wild-type (*Ttpa*^*+/+*^) mice takes many months owing to its long half-life in this tissue [18]. The *Ttpa*^*−/−*^ mouse model is an effective tool to study the impact of vitamin E status on health and disease prevention because *Ttpa*^*−/−*^ mice have low αT body stores and their tissues are more easily depleted than wild-type mice [19]. α-TTP is mainly expressed in the liver and facilitates the packaging of αT into lipoproteins to be distributed to peripheral tissues [20]. For example, the expression of genes related to oxidative stress, inflammation, myelination, neurogenesis, and innate immune response was assessed with this mouse model [5, 17, [21], [22], [23]].

LPS challenges are commonly used in mice to induce systemic inflammation and neuroinflammation, which may trigger the neurodegenerative process related to many neurological disorders [[24], [25], [26]]. Low αT status has been shown to exacerbate the oxidative stress response, such as reducing plasma glutathione peroxidase activity and increasing hepatic thiobarbituric acid reactive substances after LPS exposure in *Ttpa*^*+/+*^ mice [[27], [28]]. The peripheral innate immune system activates brain microglia to enhance inflammatory cytokine production that is associated with sickness behavior in mice. Godbout et al. [29] indicated that αT supplementation attenuated peroxide racial production and IL-6 secretion following LPS exposure in primary microglia and in the brains of mice. However, these researchers did not specify whether the vitamin E concentrations in the brain were completely depleted [[27], [28]]. Moreover, there are no data on the impact of vitamin E status on inflammatory, immune cell profiles, and oxidative stress response in the brain and heart following LPS injection in *Ttpa*^*−/−*^ mice during vitamin E depletion. For our study, we employed an LPS challenge in *Ttpa*^*−/−*^ mice and then assessed the effect of vitamin E status on markers of inflammation and oxidative stress.

The objective for this study was to examine how extremely low αT status, followed by exposure to LPS, altered the acute inflammatory response to LPS in *Ttpa*^*−/−*^ and wild-type mice. The overall hypothesis for this study was that oxidative stress would be exacerbated in *Ttpa*^*−/−*^ mice fed a vitamin E deficient (VED) diet, compared with the wild-type control group. We also hypothesized that *Ttpa*^*−/−*^ mice would demonstrate an enhanced immune response compared with *Ttpa*^*+/+*^ mice, owing to their lower vitamin E status and predicted increases in oxidative stress.

## Methods

### Animals

The University of Illinois Institutional Animal Care and Use Committee approved all animal procedures. Study mice were generated using a trio-breeder strategy [30]. Briefly, male *Ttpa*^*−/−*^ and female C57BL6 (*Ttpa*^*+/+*^) were crossed to generate *Ttpa*^*+/−*^ mice. *Ttpa*^*+/−*^ mice (1 male and 2 females/cage) were then bred to produce male *Ttpa*^*−/−*^ mice (*n* = 36) and control *Ttpa*^*+/+*^ (*n* = 36) littermates for the study. The heterozygous breeders were fed an AIN-93G-based, low vitamin E (LOW) diet [35 mg *RRR*-α-tocopherol acetate (αTA)/kg diet] to minimize brain αT accumulation in the offspring (Supplemental Table 1). The offspring genotypes were confirmed via polymerase chain reaction with specific primers for *Ttpa* as previously described [31]. This study used exclusively male mice to remove sex as a possible variable [32]. At weaning (3 wk old), study mice were randomly assigned to treatment groups and individually housed in shoebox cages (12:12-h light-dark cycle, 22°C, 60% humidity) and provided a VED diet ad libitum until study termination at 7 wk of age (4 wk feeding period) [33]. Throughout the experimental period, weekly body weight and food intake were assessed.

Diet was removed from cages on the morning of killing ∼8 h before tissue harvest. Four hours later, mice were injected either intraperitoneal LPS (Sigma cat #L4516; *Escherichia coli* O127:B8, 1 or 10 μg/mouse) or saline as control. These doses were determined based on previous studies of LPS-induced neuroinflammation in mice [[27], [28], 34]. The study mice were anesthetized with ketamine/xylazine (87 mg/mL and 13 mg/mL, respectively) as a terminal procedure and killed 4 h postinjection. A 4-h treatment with LPS was determined based on studies that were similar to our current study design [28, 34]. LPS-induced neuroinflammation has been measured 4 h after injection [34], and oxidative stress markers were upregulated in several brain regions [28, 34]. Cardiac puncture was carried out to collect blood, and serum was separated by centrifugation (Eppendorf model 5417R) (2400 × g, 10 min, 4°C). Tissues were dissected, weighed, and immediately snap-frozen in liquid nitrogen. Brain regions were further dissected to isolate the cerebral cortex, cerebellum, and hippocampus. For reverse transcriptase-quantitative PCR (RT-qPCR), select tissues were placed in RNA*later* (Thermo Fisher) before freezing. Serum and harvested tissues were stored at −80°C until further analysis.

### Diets

All animal diets were customized, and diet preparation procedures were followed as in a previous study [33]. Briefly, the VED and LOW diets were purchased from Research Diets, Inc. Modified-AIN-93G formulation, including replacing most of the soybean oil with hydrogenated coconut oil to minimize dietary concentrations of vitamin E, was used for these experiments and was based on the diets used in previous studies [33, 35]. *RRR*-αTA [Novatol 6-92 (oil); Archer Daniels Midland] was added to the LOW diet. Before diet production, the oil was assessed via HPLC with photodiode array detection (HPLC-PDA) to verify αTA purity. Both diets contained the expected αTA concentrations, as verified via HPLC-PDA [33]. The lower limit of detection for αTA in the study diet was 0.49 mg/kg diet. Diets were stored with vacuum-sealing at −20°C, and mice were provided new pellets once per week.

### Vitamin E analysis via HPLC-PDA

αT accumulation in serum and target tissues (brain, liver, heart, kidney, and lung) were measured via HPLC as previously described [33]. The αT extraction in adipose tissue was carried out using procedures detailed previously [36]. The lower limits of detection for αT were 0.11 μmol/L for serum and 0.12 nmol/g for tissues.

### Inflammatory response via ELISA

IL-6 was measured in the cerebellum, heart, and serum by ELISA (Mouse IL-6 ELISA Kit; Sigma) following the manufacturer’s instructions. Briefly, tissue samples (∼20 mg) were homogenized with 1× phosphate-buffered saline (PBS) containing 1% protease inhibitor cocktail (Sigma) and 1% Triton X-100 (Sigma) to obtain total protein extracts. Protein concentrations in the target tissues and serum were analyzed using the Pierce BCA protein assay kit (Thermo Scientific). All protein samples were run in duplicate. Serum and tissue IL-6 concentrations were analyzed by SoftMax Pro 5.2 (Molecular Devices).

### Circulating immune cell profile via Heska

The circulating immune cell profile was analyzed by Element HT5 (Heska). After blood collection, 15–20 μL of each sample was immediately measured. The number and percent of lymphocytes, monocytes, neutrophils, eosinophils, and basophils were determined. All blood samples were run in duplicate.

### Total RNA isolation and RT-qPCR analysis

Total RNA was isolated and RT-qPCR analysis was conducted using previously described procedures [35, 37]. Briefly, hippocampal and heart total RNA was isolated using RNeasy Lipid Tissue Mini Kit (Qiagen) via the manufacturer’s protocol. Complementary DNA was synthesized from total RNA isolation by RT^2^ First Strand Kit (Qiagen). RNA purities and concentrations were measured by Biospectrometer (Eppendorf) and agarose gel electrophoresis. After all RNA samples were confirmed to meet the quality control requirements, RT-qPCR was carried out using a Quant Studio 3 Real-Time PCR system (Applied Biosystems by Thermo Fisher) according to the manufacturer’s instructions for SYBR Green ROX qPCR Mastermix (Qiagen). The 2^-ΔΔCt^ method was used to calculate relative gene expression levels. Actin B1 (*Actb1*) was used as a reference gene. Genes associated with inflammation and oxidative stress were selected based on previous research [27, 35]. Primers were obtained from Integrated DNA Technologies and primer sequences are listed in Supplemental Table 2.

### Lipid peroxidation

Malondialdehyde concentration was analyzed in the heart by TBARS assay (OxiSelect TBARS Assay Kit, MDA Quantification, Cell Biolabs) following the manufacturer’s instructions. Briefly, heart samples (∼20 mg) were homogenized with 1× PBS (∼200 μL) and 1× butylated hydroxytoluene solution (Cell Biolabs). After incubation and centrifugation, butanol extraction was carried out. Malondialdehyde concentrations were measured by SoftMax Pro 5.2 (Molecular Devices).

Total 8-isoprostane concentration in the heart was measured by ELISA using the 8-isoprostane ELISA Kit (Cayman). Tissue homogenization, sample purification using a solid-phase extraction purification protocol (Cayman), and 8-isoprostane analysis were performed following the manufacturer’s instructions.

### Glial fibrillary acidic protein expression via western blot

The same protein extracts described previously (Inflammatory response via ELISA section of the Methods) were used for glial fibrillary acidic protein (GFAP) expression by western blot. All procedures of sodium dodecyl sulfate polyacrylamide gel electrophoresis, protein transfer, immunoblotting, and detection method were followed as described previously [37]. The primary antibodies and their dilutions were as follows: GFAP, 1:1000; GAPDH, 1:3000 [Cell Signaling; GFAP (D1F4Q) XP Rabbit mAb #12389 and GAPDH (D16H11) XP Rabbit mAb #5174]. The band intensities were visualized through ImageQuant LAS 4000 (GE Healthcare), and the quantification was assessed by Quantity One software (Bio-Rad).

### Statistical analysis

GraphPad Prism version 8.1.3 for Windows was used for all data analysis. Before conducting any statistical analysis, the Shapiro-Wilk and Brown-Forsythe tests were carried out to examine normality and homogeneity of variance, respectively. If these assumptions were not met on data, the following transformations were used: Y = log(Y), Y squared, or 1/Y. If the assumptions were still not met after transforming the data, the nonparametric Kruskal-Wallis test was used. For most study endpoints, differences between genotype (*Ttpa*^*−/−*^ or *Ttpa*^*+/+*^) and treatment groups (LPS 1 μg, LPS 10 μg, or saline) were assessed 2 × 3 factorial analysis of variance, followed by Tukey’s post hoc test when justified. Values are represented as mean ± standard error of the mean. Differences between experimental groups were considered significant when *P* <0.05.

## Results

### Body mass, tissue weights, and food intake

Weekly body weight increased over time for all *Ttpa*^*−/−*^ and *Ttpa*^*+/+*^ mice (*P* < 0.0001); however, there were no significant differences in body weight at weaning, during experimental periods, and at termination between the genotypes or treatment groups (*P* = 0.71). Tissue weights did not differ between groups (data not shown). Additionally, there was no difference in food intake between groups (*P* = 0.70), but there was a significant time effect for increased average weekly food consumption for all mice (*P* < 0.0001) (Supplemental Figure 1).

### αT analysis

There was no change in αT tissue concentrations due to LPS exposure (Supplemental Table 3); therefore, we combined the 2 genotype groups (*Ttpa*^*−/−*^ vs. *Ttpa*^*+/+*^) for statistical evaluation of αT content in serum and the target tissues. Serum and selected tissue αT accumulations in *Ttpa*^*−/−*^ mice were substantially lower than *Ttpa*^*+/+*^ mice (Table 1). αT accumulation in serum, the liver, and adipose tissue was statistically higher in *Ttpa*^*+/+*^ mice than *Ttpa*^*−/−*^ mice (*P* < 0.0001). αT was not detected in the brain or heart of *Ttpa*^*−/−*^ mice, whereas *Ttpa*^*+/+*^ mice accumulated αT in the brain and heart and had higher αT in the other measured tissues. Other peripheral tissue αT concentrations are shown in Supplemental Table 4.

**TABLE 1:** αT concentrations in serum and selected tissues of *Ttpa*^*−/−*^ and *Ttpa*^*+/+*^ mice.

Genotype/tissue	Pooled serum	Liver	Heart	Adipose tissue	Brain
*Ttpa*^*+/+*^	3.0 ± 0.5^1^	7.5 ± 0.4^1^	16.4 ± 1.4	13.6 ± 0.6^1^	15.6 ± 0.2
*Ttpa*^*−/−*^	0.16 ± 0.008^2^	2.0 ± 0.3^2^	ND	0.9 ± 0.04^2^	ND

αT, α-tocopherol; ND, not detected; *Ttpa*, α-tocopherol transfer protein.

Results in pooled serum and tissues are shown as mean ± SEM μmol/L (*n* = 3/genotype) or nmol/g (*n* = 9/genotype), respectively. ND, Lower limits of detection: 0.11 μmol/L (serum); 0.12 nmol/g (tissues). Different superscript numerals denote significant differences (*P* < 0.0001) between genotypes by a 2-tailed Student’s *t* test.

### Inflammatory response and oxidative stress measurements in brain, heart, and serum

**FIGURE 1:**
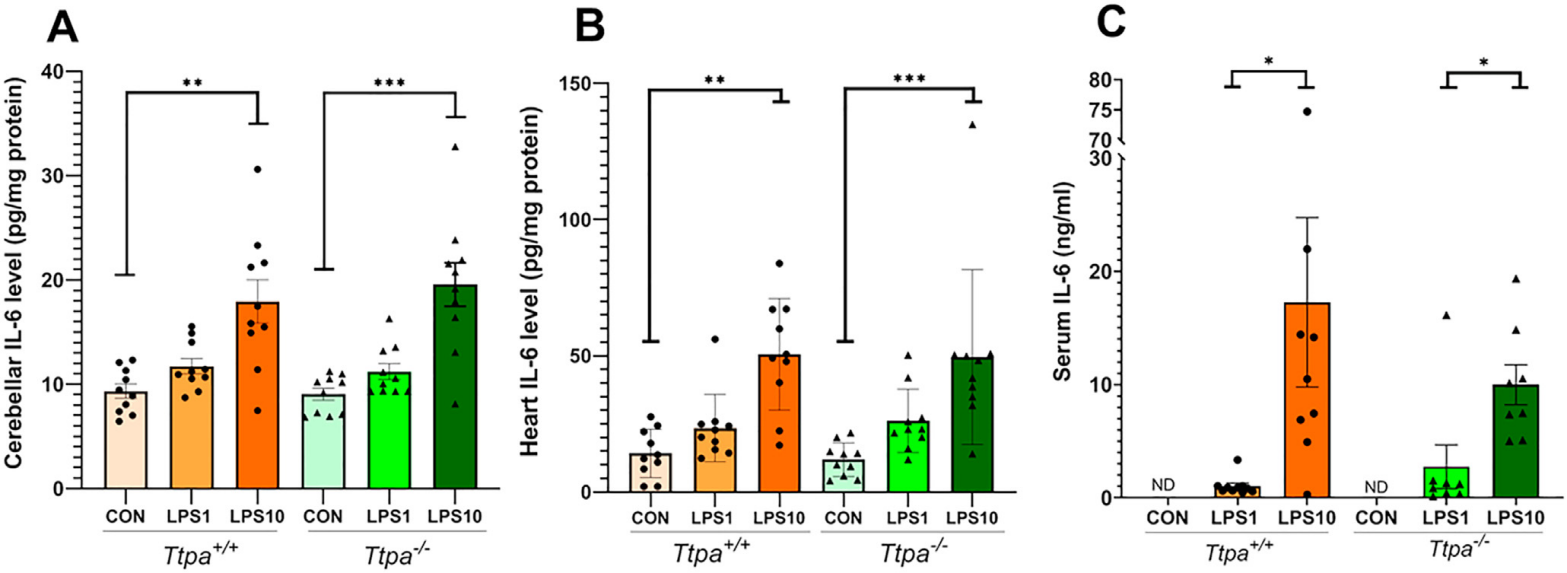
IL-6 concentrations in (A) cerebellum; (B) heart; and (C) serum in *Ttpa*^*−/−*^ and *Ttpa*^*+/+*^ mice (*n* = 10/tissue/group and *n* = 8–10/serum/group). All values are represented as mean ± SEM ng/mL (serum) or pg/mg protein (tissues), respectively. Nonparametric Kruskal-Wallis test with Dunn’s multiple comparison test was conducted. Each circle and triangle express the measured value from an individual animal. Significance: ∗ *P* < 0.05. ∗∗ *P* < 0.01. ∗∗∗ *P* < 0.001. Lower limit of detection: 2 pg/mL. CON, control; ND, not detected; *Ttpa*, α-tocopherol transfer protein.

Serum, cerebellum, and heart IL-6 concentrations were measured in *Ttpa*^*+/+*^ and *Ttpa*^*−/−*^ mice 4 h after injection of LPS or saline (Figure 1). The 10 μg LPS groups had increased cerebellar and cardiac IL-6 concentrations compared with controls, confirming an acute inflammatory response (*P* < 0.01) (Figure 1A and 1B). However, there was no significant difference between genotypes in these tissues. In serum, there was a significant difference between LPS 1 μg and 10 μg groups (*P* < 0.05) (Figure 1C). Serum IL-6 concentrations in the control groups were below assay sensitivity. Comparing LPS effects by genotype, there was no statistically significant difference between the study groups.

To assess oxidative stress levels, malondialdehyde and 8-isoprostane concentrations in the heart were examined. There was no significant change in malondialdehyde or 8-isoprostane concentrations by LPS exposure in the heart between treatment groups (Supplemental Table 5).

### Circulating immune cell profile

White blood cell concentration in the blood, particularly lymphocytes, were decreased in all LPS treatment groups compared with the saline control groups (*P* < 0.01) (Table 2). White blood cell counts were decreased by LPS exposure (*P* < 0.001), but there were no genotype effects or the interaction between LPS treatment and genotypes (*P* = 0.09 and *P* = 0.17, respectively). The pattern of lymphocytes, monocytes, and basophils reflected that of total white blood cells within treatment groups. The circulating basophil concentration in 1 μg LPS-*Ttpa*^*−/−*^ mice was significantly lower than in control *Ttpa*^*−/−*^ mice (*P* = 0.017), but there were no significant differences in basophil concentrations across the other experimental groups. Although there was a significant change following exposure to LPS in circulating immune cells, there were no significant differences by genotype for any of the measured circulating immune cell types.

**TABLE 2:** Blood immune cell numbers in *Ttpa*^*−/−*^ and *Ttpa*^*+/+*^ mice 4 h after injection of LPS or saline.

Cell type	*Ttpa*^*+/+*^			*Ttpa*^*−/−*^		
	CON	LPS 1	LPS 10	CON	LPS 1	LPS 10
White blood cells	3.19 ± 0.25^1^	1.54 ± 0.18^2^	1.80 ± 0.19^2^	3.57 ± 0.29^1^	2.13 ± 0.21^2^	1.75 ± 0.24^2^
Neutrophils	0.89 ± 0.14	0.71 ± 0.09	0.81 ± 0.10	0.89 ± 0.11	1.16 ± 0.18	0.88 ± 0.10
Lymphocytes	2.02 ± 0.17^1^	0.70 ± 0.08^2^	0.81 ± 0.09^2^	2.35 ± 0.22^1^	0.83 ± 0.06^2^	0.71 ± 0.14^2^
Monocytes	0.17 ± 0.02^1^	0.06 ± 0.01^2^	0.08 ± 0.01^1^^,^^2^	0.16 ± 0.03^1^	0.07 ± 0.02^2^	0.08 ± 0.02^2^
Eosinophils	0.06 ± 0.01	0.06 ± 0.02	0.07 ± 0.02	0.08 ± 0.02	0.05 ± 0.02	0.06 ± 0.02
Basophils	0.05 ± 0.03^1^^,^^2^	0.02 ± 0.01^2^	0.03 ± 0.01^1^^,^^2^	0.09 ± 0.04^1^	0.01 ± 0.01^2^	0.02 ± 0.01^1^^,^^2^

CON, control; *Ttpa*, α-tocopherol transfer protein. Each value is shown as mean ± SEM 10^3^/μL (*n* = 12/treatment group). Different superscript numericals denote significant differences (*P* < 0.05) between LPS treatment groups by 2 × 3 factorial analysis of variance with Tukey’s post hoc test or nonparametric Kruskal-Wallis test with Dunn’s multiple comparison test.

### The expression of genes associated with inflammation and oxidative stress

**FIGURE 2:**
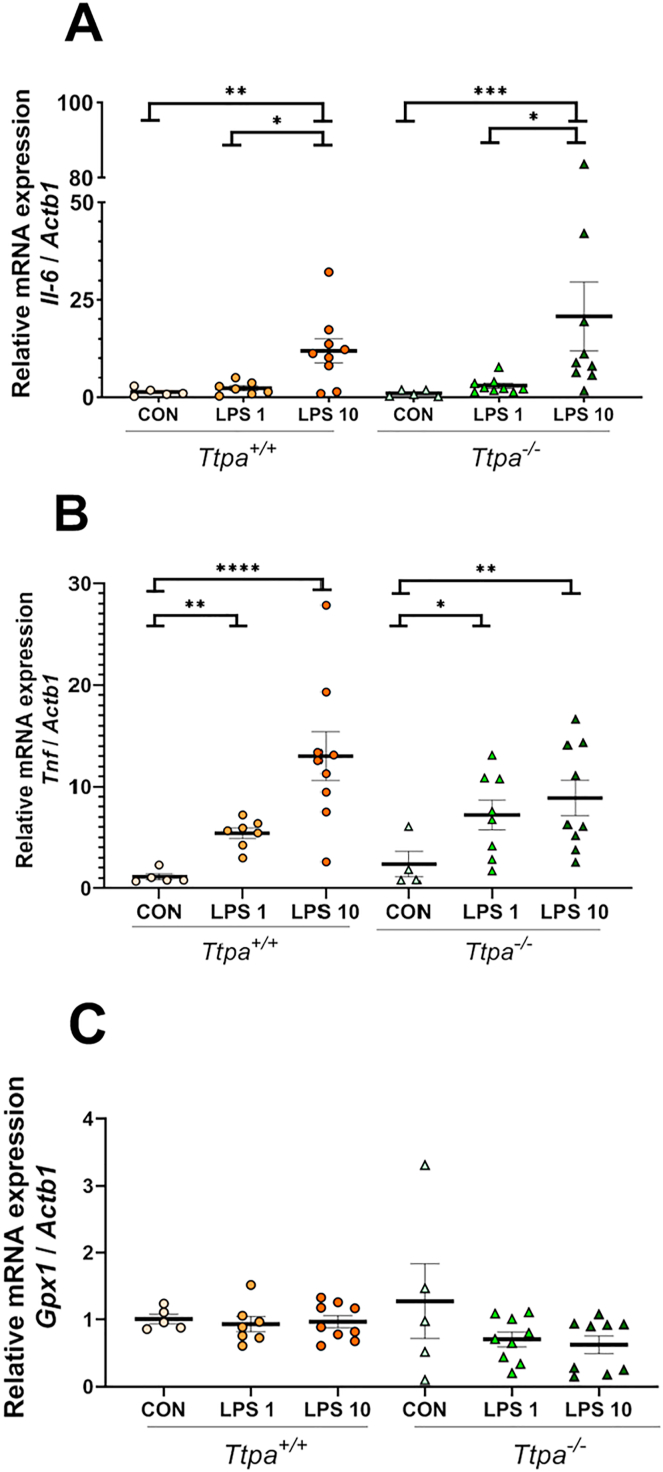
Hippocampal (A) *Il-6*; (B) *Tnf*; and (C) *Gpx1* expression in *Ttpa*^*−/−*^ and *Ttpa*^*+/+*^ mice. Results are shown as mean ± SEM (*n* = 4–9/group). 2 × 3 factorial ANOVA with Tukey’s post hoc test or nonparametric Kruskal-Wallis test with Dunn’s multiple comparison test were conducted. Each circle and triangle express the measured value from an individual animal. Significance: ∗ *P* < 0.05. ∗∗ *P* < 0.01. ∗∗∗ *P* < 0.001. ∗∗∗∗ *P* < 0.0001. CON, control; ND, not detected; *Ttpa*, α-tocopherol transfer protein.

**FIGURE 3:**
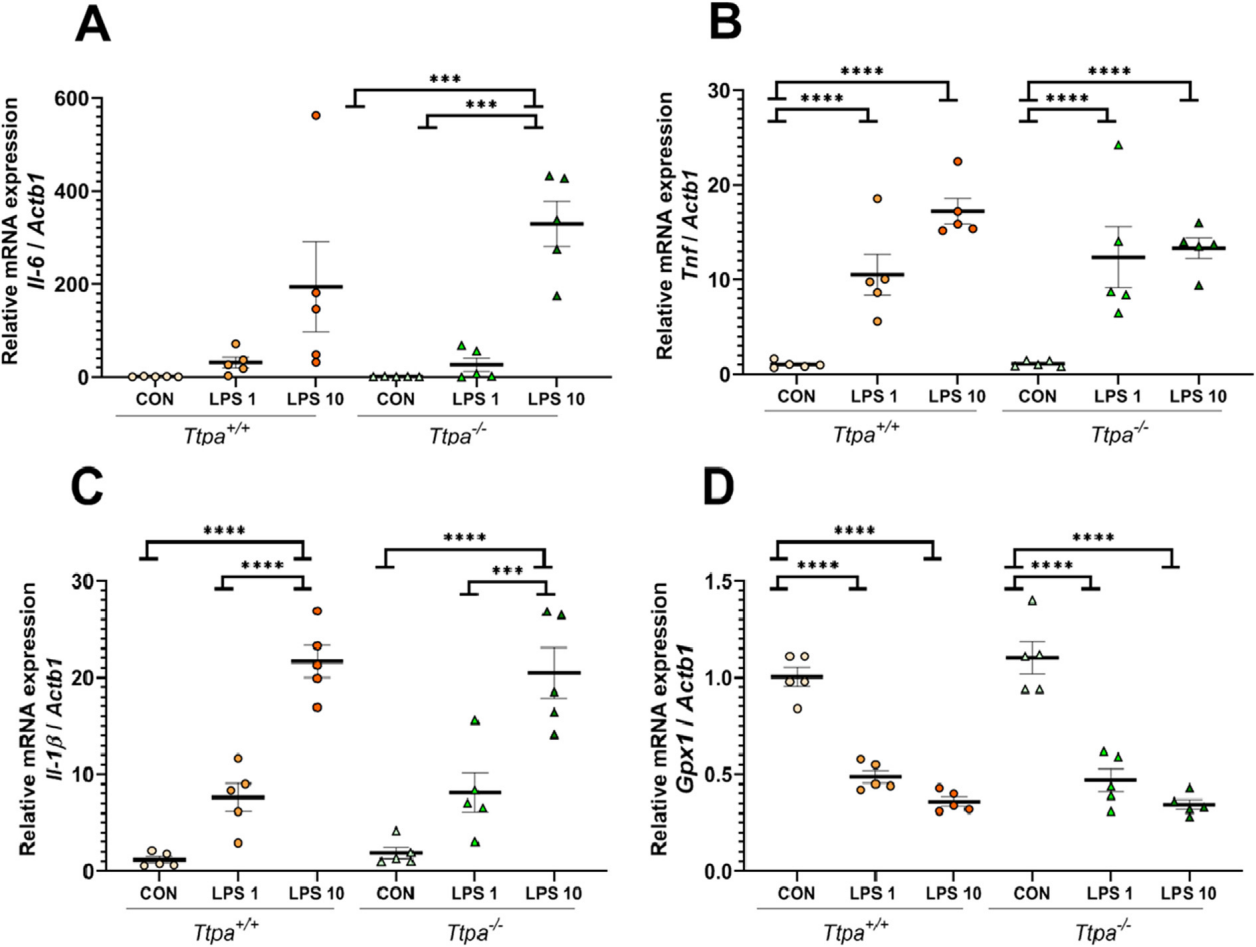
Heart (A) *Il-6*; (B) *Tnf*; (C) *Il-1β*; and (D) *Gpx1* expression in *Ttpa*^*−/−*^ and *Ttpa*^*+/+*^ mice. Results are shown as mean ± SEM (*n* = 5/group). 2 × 3 factorial ANOVA with Tukey’s post hoc test was conducted. Each circle and triangle express the measured value from an individual animal. Significance: ∗ *P* < 0.05. ∗∗ *P* < 0.01. ∗∗∗ *P* < 0.001. ∗∗∗∗ *P* < 0.0001. CON, control; ND, not detected; *Ttpa*, α-tocopherol transfer protein.

The gene expression of *Il-6*, *Tnf*, *Il-1β*, and *Gpx1*, markers of inflammation and oxidative stress, was analyzed in the hippocampus and heart of *Ttpa*^*+/+*^ and *Ttpa*^*−/−*^ mice (FIGURE 2, FIGURE 3). Hippocampal *Il-6* expression was significantly upregulated in the 10 μg LPS groups compared with the control and 1 μg LPS groups (*P* < 0.05) (Figure 2A). *Tnf* gene expression was also increased in both LPS groups (1 and 10 μg LPS) compared with control groups, suggesting a neuroinflammatory response at the transcriptional level (*P* < 0.05) (Figure 2B). *Il-6* expression in the hippocampus was upregulated by LPS in a dose-dependent manner (*P* < 0.05), indicating that the higher dose of LPS (10 μg LPS/mouse) upregulated *Il-6* gene expression more than a lower dose of LPS (1 μg) or control (0 μg). There was no change in hippocampal *Gpx1* gene expression between the treatment groups (Figure 2C). For the comparison of expression patterns by genotype, although the 10 μg LPS-*Ttpa*^*−/−*^ mice had ∼1.6-fold lower *Gpx1* and ∼1.7-fold higher *Il-6* expression than the 10 μg LPS-*Ttpa*^*+/+*^ mice, these genotype differences were not statistically significant.

In the heart, *Il-6* expression was significantly higher in the 10 μg LPS-*Ttpa*^*−/−*^ mice than LPS 1 μg- and control-*Ttpa*^*−/−*^ mice (*P* < 0.001), but there were no differences between the *Ttpa*^*+/+*^ mouse groups as shown in Figure 3A. *Tnf* expression in the heart was also upregulated in the LPS-treated groups compared with the saline control groups (*P* < 0.0001) (Figure 3B). Heart *Il-1β* gene expression was increased by LPS in a dose-dependent manner (*P* < 0.001) (Figure 3C), showing higher LPS exposure enhanced production of this cytokine more than lower/no LPS exposure. However, heart *Il-6, Il-1β,* and *Tnf* gene expressions between LPS-treated *Ttpa*^*−/−*^ and *Ttpa*^*+/+*^ mice were not significantly different. Although hippocampal *Gpx1* gene expression was not affected by LPS exposure, the gene expression in heart was significantly downregulated in LPS groups compared with control groups (*P* < 0.0001) (Figure 3D). There were no interactive effects between LPS treatment and genotypes for the measured genes in the hippocampus and heart (*P* > 0.20).

### GFAP expression in cerebellum

GFAP expression was measured via western blot in the cerebellum and spinal cord to assess astroglia activation following LPS exposure. There was no change between genotypes or among LPS treatments in either tissue, although GFAP in the spinal cord was much more highly expressed compared with the cerebellum (data not shown).

## Discussion

The objective of this study was to utilize a transgenic mouse model (*Ttpa*^*−*/*−*^) to deplete vitamin E in tissues, stress the mice with LPS, and then assess the effects of very low vitamin E status on acute inflammatory and oxidative stress markers. LPS dosing induced inflammatory cytokine stimulation in the analyzed tissues, largely in a dose-dependent manner. There was a trend (ns) for enhanced expression in genes related to inflammatory and oxidative stress response in the hippocampus and heart in LPS-exposed *Ttpa*^*−/−*^ mice. However, there were no significant additional effects seen comparing *Ttpa*^*−/−*^ to *Ttpa*^*+/+*^ mice on measured markers of the inflammatory and oxidative stress response despite even lower vitamin E status and αT depletion in many *Ttpa*^*−*/*−*^ mouse tissues. We initially hypothesized that the 4-wk vitamin E–depletion period in *Ttpa*^*−/−*^ mice (7 wk old) would be sufficient to enhance sensitivity to inflammation and oxidative stress compared with wild-type mice. However, there were no substantial differences in IL-6 protein expression in the cerebellum, heart, and serum, or in the expression of any of the selected genes between *Ttpa*^*+/+*^ and *Ttpa*^*−/−*^ mice.

We chose to focus on the brain and heart for our analyses, as an abundance of research supports vitamin E’s antioxidant role in these tissues [3, 31, 35, 38]. The correlation between cardiovascular disease/immune systems and αT has been examined in epidemiological, animal, and human studies [39]. αT modestly decreased expression of genes related to the inflammatory response in human cell lines, suggesting that αT has an important role in anti-inflammation in the heart [38]. The cerebellum and the hippocampus are potential brain regions that are sensitive to neuroinflammation. Rota et al. [40] showed that αT modulated hippocampal gene expression related to Parkinson’s disease and Alzheimer’s disease in rats. In the current study, we showed enhanced inflammatory response in the hippocampus and cerebellum.

In the current study, genotype groups were fed a VED diet, and study results showed that *Ttpa*^*+/+*^ mice maintained some αT in tissues and serum over this time period [33, 35]. In *Ttpa*^*−/−*^ mice, serum and some peripheral tissues, such as liver and adipose tissue, were not completely devoid of vitamin E whereas in other tissues vitamin E was depleted. Our previous study showed that a 4-wk vitamin E–depletion period in *Ttpa*^*−/−*^ mice was sufficient to deplete αT in serum and most tissues except the liver, but that study had a slightly different breeding protocol [33]. Ranard et al. (2020) fed *Ttpa* heterozygous breeders with a VED diet beginning at day 9 of the gestation period through lactation to minimize transport of αT to their offspring [33]. In contrast, our breeders received a LOW diet (30 mg d-αTA/kg diet) during gestation and lactation periods. The latter breeding protocol may alter the transferred αT to offspring, but overall αT accumulation should not be markedly different.

According to several *Ttpa*^*−/−*^ mouse model studies, vitamin E deficiency induced downregulation of transcription factors related to myelination and synaptogenesis and enhanced oxidative stress and abnormal behavior functions in young and aged *Ttpa*^*−/−*^ mice [[21], [22]]. However, relatively few studies have examined the effects of vitamin E status on LPS-induced oxidative stress and inflammatory responses in *Ttpa*^*−/−*^ mice. Chung et al. [41] have shown that αT supplementation contributed to reduce LPS-induced oxidative stress and inflammatory markers, such as hepatic malondialdehyde and TNF-α concentrations, in obese *Ttpa*^*−/−*^ mice, confirming the attenuated inflammatory response 6 h postintraperitoneal LPS injection (250 μg/kg, single dose). Schock et al. [24] also demonstrated LPS-induced oxidative stress and inflammation in plasma, liver, and lung of *Ttpa*^*−/−*^ mice 12 h postinjection (LPS 10 mg/kg intraperitoneally), although the results did not show significant differences of lower vitamin E status on the inflammatory-immune response in *Ttpa*^*−/−*^ mice compared to wild-type mice. LPS triggers not only an oxidative stress response; it could affect many signaling and gene expression pathways via Toll-like receptor. Other pathways may well be impacted by LPS. LPS stimulates TLR4 by enhancing the innate immune system, which is induced by pathogen-associated molecular patterns [42]. LPS-induced TLR4 induces production of pro-inflammatory cytokines. Although vitamin E supplementation enhanced recovery from LPS-induced sickness behavior and cytokine production in mice [28], αT itself did not directly activate the TLR4 pathway [43].

LPS-induced inflammatory responses robustly upregulated *Il-1β, Il-6,* and *Tnf* mRNA concentrations in the hippocampus and heart of 7-wk-old mice 4 h after injection. Some studies have shown that a single dose of LPS rapidly induces pro-inflammatory cytokine gene expression in the brain at 2 h postinjection [[44], [45]]. Similarly, our study also indicated a significant difference in neuroinflammatory response between LPS and control groups. Although there was no difference in hippocampal *Gpx1* gene expression, a marker of the oxidative stress response, between treatment groups, heart *Gpx1* gene expression was significantly downregulated in the LPS groups, confirming oxidative stress response at the transcriptional level in that tissue.

We report here altered blood immune cell profiles, suggesting an acute inflammatory response in these cells. We had hypothesized that immune cell numbers would increase following LPS-induced inflammation. However, the results showed that white blood cells, specifically lymphocytes and monocytes, were decreased at this time point. This suggests that the immune response rapidly occurred, and that immune cells most likely moved into peripheral tissues. These findings will inform future studies in which we aim to optimize our mouse model to establish more reliable timing for measuring markers of neuroinflammation and oxidative stress in *Ttpa*^*−/−*^ mice. The 4 wk of dietary vitamin E restriction may not have been sufficient to reduce vitamin E status significantly in LPS-induced oxidative stress and inflammation compared with wild-type mice. In a follow-up study, we plan to use an 8-wk αT depletion period to evaluate the immune response.

Many studies have shown the LPS effects on oxidative stress pathways in lipid-rich tissues and cytokine production in peripheral tissues and circulation [24, [27], [28], [29], 34, 44]. Dietary vitamin E restriction in our study did not affect antioxidant markers in the heart (malondialdehyde and 8-isoprostane concentrations). In contrast, Berg et al. [27] reported decreased plasma glutathione peroxidase activity and increased hepatic thiobarbituric acid reactive substances in 3- to 6-month-old wild-type mice fed a low vitamin E diet (10 mg αTA/kg diet) compared with mice fed a high vitamin E diet (500 mg αTA/kg diet). Another study showed that LPS injections induced intracellular peroxides and increased supernatant IL-6 concentrations in primary microglia (the brain’s resident immune cells) in 3-month-old mice. This shows that a high dose of LPS can rapidly stimulate an oxidative stress response [29]. Those researchers also indicated that αT treatments decreased peroxide radical synthesis and IL-6 secretion in microglia, suggesting that supplemental αT may affect neuroimmune function and prevent oxidative stress in the brain [29].

The difference between the results of previous studies and the current study may be due to the short time between LPS dose and tissue evaluation, reflecting an acute inflammatory response. We harvested tissues 4 h after the LPS challenge, whereas most other researchers collected samples 24 h postinjection. Thus, the oxidative stress response to LPS may require >4 h following LPS injection. Furthermore, previous studies used relatively higher LPS doses, such as 100 μg/mouse, which is 10 times higher than the highest dose in our study. The higher dose presumably would cause higher inflammation and oxidative stress in mice. Moreover, the exact composition of LPS and/or source may cause adverse results compared with previous findings.

LPS-induced neural deficits may also include the dysregulation of brain specific proteins, such as GFAP. This protein is used as a cell-specific marker for astrocytes as it facilitates efflux of vitamin E to neighboring neurons [8] and may be important for CNS myelin development. It has been demonstrated that LPS exposure increases GFAP protein expression in the cerebral cortex and spinal cord of mice and rats, respectively [46, 47]. Enhanced GFAP protein expression leads to astrocyte activation, and these activated neuroglial cells promoted the secretion of inflammatory cytokines [47]. Thus, GFAP may be an effective neurobiomarker for neurological disorders, specifically gliosis [48]. Although a ∼40% reduction in *GFAP* gene expression was reported in the brain cortex of 10-month-old *Ttpa*^*−/−*^ mice [22], suggesting that vitamin E status may modulate GFAP, in the current study, GFAP protein expression was not altered by LPS exposure. Most likely there was an insufficient period after intraperitoneal injection to increase GFAP expression in the brain.

In conclusion, the present study demonstrated that the higher LPS dose elevated the acute inflammatory markers in the brain, heart, and serum in both *Ttpa*^*−/−*^ and wild-type mice to a similar degree, despite their differing vitamin E status. The lack of genotype effect in the present study may be because of the relatively young age of *Ttpa*^*−/−*^ mice, the LPS dose chosen, the length of vitamin E depletion, and/or the time selected for post-LPS dose end point evaluation. Further studies are needed to further optimize the vitamin E depletion model.

## Author disclosures

MH, KMR, AJS, and JWE declare no conflicts of interest. Abbott Nutrition had no role in the design, implementation, analysis, and interpretation of the data.

## Data Availability

Data described in the manuscript will be made available upon request once the manuscript is published.

## Appendix A Supplementary data

Multimedia components 1 Multimedia components 1
