# Supplementary material for: α-Tocopherol Transfer Protein-Null Mice with Very Low α-Tocopherol Status Do Not Have an Enhanced Lipopolysaccharide-Induced Acute Inflammatory Response
Source: Curr Dev Nutr. 2023 Jan 13;7(1):100017. doi: 10.1016/j.cdnut.2022.100017 (PMC10100938; doi:10.1016/j.cdnut.2022.100017)
Supplement: Multimedia components 1 [file mmc1.docx]

**Supplementary Material**

**Supplemental Table 1.** Modified AIN-93G basal diet composition and content of α-tocopherol

acetate added to experimental diets.^1^

| **Ingredient (g/kg diet)** | **VED** | **LOW** |
| --- | --- | --- |
| Casein | 200 | 200 |
| L-Cystine | 3 | 3 |
| Corn starch | 398 | 398 |
| Maltodextrin | 132 | 132 |
| Sucrose | 100 | 100 |
| Cellulose | 50 | 50 |
| Soybean oil^2^ | 25.9 | 25.9 |
| Hydrogenated coconut oil^3^ | 44.1 | 44.1 |
| t-butylhydroquinone | 0.01 | 0.01 |
| Mineral mix^4^ | 35 | 35 |
| Vitamin mix^5^ | 10 | 10 |
| Choline bitartrate | 2.5 | 2.5 |
| Novatol oil (86 % *RRR*-αT)^6^ (mg/kg) | 0 | 0.035 |
| **Total αTA (mg/kg diet)** | ND^7^ | 36.6 |

^1^Diets were prepared by Research Diets (New Brunswick, NJ)

^2^Incorporated at levels necessary to meet essential fatty acid recommendations for rodents. Soybean oil analysis: estimated natural αT added from soybean oil: ~2 mg/kg diet.

^3^αT was undetectable in manufacturer analysis (Dyets, Bethlehem, PA)

^4^AIN-93 mineral mix for growing rodents (S10022G).

^5^AIN-93 vitamin mix with no vitamin E (V13402).

^6^The purity of αT in the oil was analyzed via HPLC before diet production

^7^αTA levels were below the limit of detection (0.49 mg/kg) in the VED diet

Abbreviations: αT, α-tocopherol; αTA, α-tocopherol acetate; LOW, low vitamin E diet; VED, vitamin E-deficient diet

**Supplemental Table 2.** Primer sequences for RT-qPCR analysis in hippocampus and heart of *Ttpa^+/+^* and *Ttpa^-/-^* mice.^1^

| **Gene symbol** | **Accession ID** | **Forward** | **Reverse** |
| --- | --- | --- | --- |
| *Actb* | NM_007393 | GGCTGTATTCCCCTCCATCG | CCAGTTGGTAACAATGCCATGT |
| *Gpx1* | NM_008160 | AGTCCACCGTGTATGCCTTCT | GAGACGCGACATTCTCAATGA |
| *Il-1β* | NM_008361.4 | CAACCAACAAGTGATATTCTCCATG | GATCCACACTCTCCAGCTGCA |
| *Il-6* | NM_031168 | CTTCCATCCAGTTGCCTTCTTG | AATTAAGCCTCCGACTTGTGAAG |
| *Tnf* | NM_013693 | CTTCTGTCTACTGAACTTCGGG | CAGGCTTGTCACTCGAATTTTG |

^1^Primer sequences (5′ to 3′) were selected using previous publications, PrimerBank, and the IDT RealTime qPCR Assay tool.


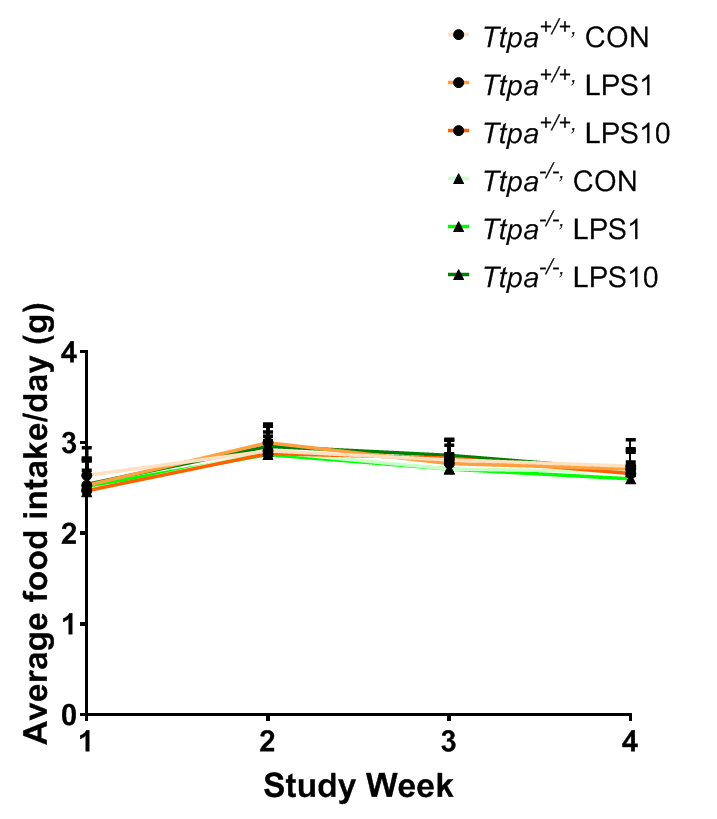


(B)

**Supplemental Figure 1. Weekly body mass (A) and average food intake/day (B) of *Ttpa^+/+^* and *Ttpa^-/-^* mice over the 4-week study period. Values are expressed as mean ± SEM. There were no significant differences between genotypes or treatment groups for body mass or average food intake, assessed by repeated measures 2-way ANOVA (n = 12 / group). Abbreviations: CON, control; LPS, lipopolysaccharide; Ttpa, α-tocopherol transfer protein.**


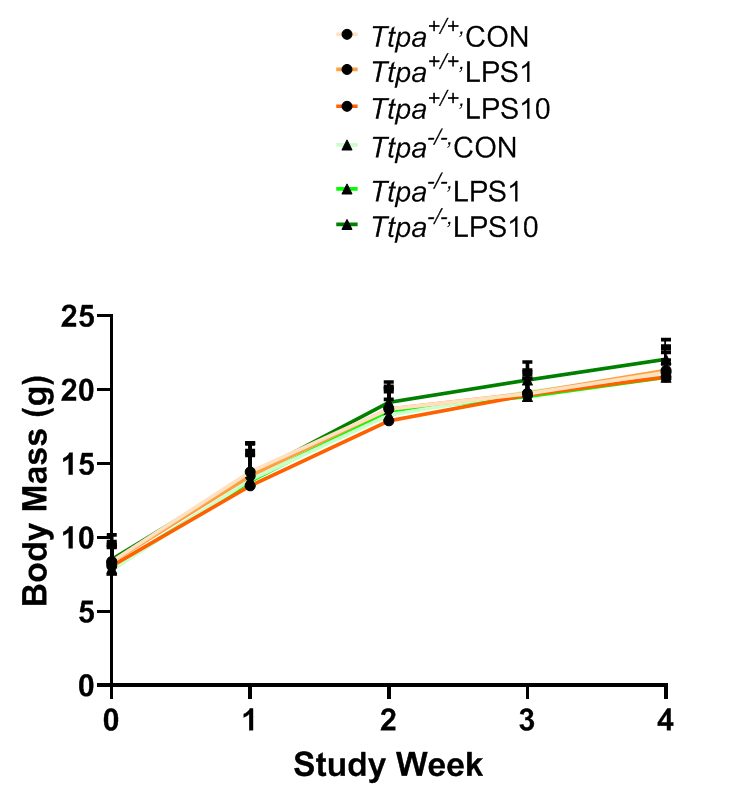


(A)

**Supplemental Table 3.** αT concentrations in serum and selected tissues between treatment groups.^1^

|  | *Ttpa^+/+^* mice | | | *Ttpa^-/-^* mice | | |
| --- | --- | --- | --- | --- | --- | --- |
| **Tissue / Group** | **CON** | **LPS 1** | **LPS 10** | **CON** | **LPS 1** | **LPS 10** |
| Pooled serum | 2.13 | 3.62 | 3.32 | 0.18 | 0.15 | 0.16 |
| Liver | 6.9 ± 0.2^a^ | 8.4 ± 0.5^a^ | 7.3 ± 1.2^a^ | 2.4 ± 0.7^b^ | 1.5 ± 0.1^b^ | 2.2 ± 0.8^b^ |
| Heart | 16.1 ± 3.2 | 14.1 ± 2.4 | 18.7 ± 1.2 | ND^2^ | ND^2^ | ND^2^ |
| Adipose tissue | 13.7 ± 1.1^a^ | 12.3 ± 0.5^a^ | 14.9 ± 1.4^a^ | 0.95 ± 0.01^b^ | 0.96 ± 0.06^b^ | 0.87 ± 0.11^b^ |
| Brain | 15.7 ± 0.03 | 15.2 ± 0.76 | 16.0 ± 0.06 | ND^2^ | ND^2^ | ND^2^ |

^1^Values are expressed as mean *±* SEM (n = 3/group). For each tissue type, different superscript letters denote significant differences (P < 0.0001) between LPS treatment groups by 2x3 Factorial ANOVA with Turkey’s post hoc test. Although there were a significant difference in αT concentrations in the liver and adipose tissue between genotypes (P < 0.0001), there was no change in hepatic and adipose tissue αT accumulation between LPS treatment groups (P = 0.90 and P = 0.37, respectively) or the interaction between LPS treatment and genotypes (P = 0.24 and P = 0.36, respectively).

^2^ ND, Lower limits of detection: 0.11 µmol/L (serum); 0.12 nmol/g (tissues).

Abbreviations: CON, control; LPS, lipopolysaccharide; Ttpa, α-tocopherol transfer protein.

**Supplemental Table 4.** αT concentrations in the kidney and lung of Ttpa^+/+^ and Ttpa^-/-^ mice.^1^

| **Genotype / Tissue** | **Kidney** | **Lung** |
| --- | --- | --- |
| *Ttpa^+/+^* | 11.8 ± 0.67^a^ | 15.8 ± 0.95^a^ |
| *Ttpa^-/-^* | 0.16 ± 0.06^b^* | 0.24 ± 0.12^b^* |

^1^Values are expressed as mean *±* SEM (n = 3/group). For each tissue type, different superscript letters denote significant differences (P < 0.0001) between genotypes by a two-tailed Student’s *t* test.

*Lower limits of detection: 0.12 nmol/g (tissues). Four and Five *Ttpa* mice had very low but detectable concentrations (<2 nmol/g) in the kidney and lung, respectively.

Abbreviations: *Ttpa,* α-tocopherol transfer protein.

**Supplemental Table 5.** MDA and 8-isoprotane levels in the heart of Ttpa^+/+^ and Ttpa^-/-^ mice.^1^

|  | *Ttpa^+/+^* | | | *Ttpa^-/-^* | | |
| --- | --- | --- | --- | --- | --- | --- |
| Gene symbol | **CON** | **LPS1** | **LPS10** | **CON** | **LPS1** | **LPS10** |
| *MDA* | 1.06 ± 0.12 | 1.20 ± 0.11 | 1.12 ± 0.10 | 1.01 ± 0.05 | 1.16 ± 0.07 | 1.15 ± 0.07 |
| *8-iso* | 86.4 ± 40.8 | 38.2 ± 7.18 | 51.3 ± 25.0 | 58.6 ± 27.1 | 54.3 ± 20.8 | 79.5 ± 29.7 |

^1^ Values are expressed as mean *±* SEM (n = /group). 2x3 Factorial ANOVA was conducted to measure differences between groups.

Abbreviations: CON, control; LPS, lipopolysaccharide; *MDA*, malondialdehyde; *Ttpa,* α-tocopherol transfer protein; 8-iso, 8-isoprostane.
